# Interactions between cancer-associated fibroblasts and the extracellular matrix in oesophageal cancer

**DOI:** 10.1016/j.matbio.2025.05.003

**Published:** 2025-05-14

**Authors:** Subashan Vadibeler, Shannique Clarke, Su M. Phyu, Eileen E. Parkes

**Affiliations:** a Centre for Immuno-Oncology, Nuffield Department of Medicine, University of Oxford, United Kingdom; b Department of Oncology, University of Oxford, United Kingdom

**Keywords:** Cancer-associated fibroblasts, Extracellular matrix, Fibrosis, Oesophageal adenocarcinoma, Oesophageal cancer, Oesophageal squamous cell carcinoma, Tumour stroma

## Abstract

Stromal components of the tumour microenvironment, such as cancer-associated fibroblasts (CAFs) and the extracellular matrix (ECM), are actively involved in tumorigenesis. CAFs and the ECM co-evolve with resultant molecular and mechanical pressure on tumour cells mediated by CAFs via the ECM. Meanwhile, ECM fibers determine CAF differentiation and activity, establishing a protumorigenic feed-forward loop. Oesophageal cancer carries a high morbidity and mortality, and curative surgical resection is only an option for a limited number of patients while early lymphatic spread and poor therapeutic responses are common. Although studies report marked heterogeneity in investigation of the stromal density of gastrointestinal cancers, it is generally accepted that oesophageal cancer is highly fibrotic, and stromal components like CAFs may outnumber cancer cells. Therefore, a comprehensive understanding of the reciprocal interaction between CAFs and the ECM in oesophageal cancer is essential to improving diagnostics and prognostication, as well as designing innovative anti-cancer strategies. Here, we summarise current understanding of oesophageal cancer from a stromal perspective. Then, we discuss that CAFs and the ECM in oesophageal cancer can independently and synergistically contribute to tumour progression and therapeutic resistance. We also summarise potential stromal targets that have been described in transcriptomic analyses, highlighting those validated in downstream experimental studies. Importantly, clinical translation of stromal-targeting strategies in oesophageal cancer is still in its infancy but holds significant promise for future therapeutic combinations.

## Introduction to oesophageal cancer

The oesophagus is a mechanical, fibromuscular tube that extends from the laryngopharynx to the gastro-oesophageal junction, with a luminal mucosal surface that constitutes of non-keratinised stratified squamous cell epithelium. Oesophageal squamous cell carcinoma (OSCC) and oesophageal adenocarcinoma (OAC) are the commonest subtypes of oesophageal cancer (OC) [1–3]. Despite the near indistin-guishable clinical presentation, the two cancer subtypes occur at different sites along the oesophagus − OSCC and OAC typically affecting the upper two-thirds and the lower third oesophagus respectively − and share very little similarity in terms of the precursor lesion, epidemiology, risk factors and treatment outcomes. Importantly, there is strong evidence to suggest that OAC may be more resistant to chemoradiotherapy [4] and immunotherapy [5,6] than OSCC, the reasons for which are only partly known, and the contribution of the stromal microenvironment is being increasingly considered as a key knowledge gap.

OC is the 11th most common cancer worldwide [7] and 14th in the UK [8]. Although OSCC is more common in low and middle-income countries [9] due to higher rates of smoking and alcohol consumption [10], OAC rates have superseded OSCC in high-income Western countries [11,12]. The UK has the highest age-standardised incidence of OAC in the world and OAC is the fastest rising cancer measured by incidence in the UK. This rise in Western OAC rates, especially among elderly Caucasian men, although also increasing in <50 year olds [13], is postulated to follow an increase in OAC risk factors such as obesity and gastro-oesophageal reflux disease. It is well-established that OAC frequently arises from or is found in conjunction with Barrett’s oesophagus [14], an acquired metaplastic transdifferentiation of squamous epithelium to an intestinalised mucosa in the lower oesophagus secondary to long-term acid reflux.

OC carries a dismal prognosis, with only 1 in 10 patients expected to survive more than 10 years [8]. Nonetheless, patient survival has more than tripled since the 1970s due to improvements in surgical intervention, especially for patients with locally advanced disease [15]. It is however widely recognised that surgical resection alone is insufficient for the management of OC due to high recurrence rates, especially for OAC [16]. Accordingly, current guidelines recommend neoadjuvant chemotherapy or chemoradiotherapy prior to surgical resection, first established in the MAGIC trial [17–20]. In the era of immunotherapy, the landmark study to date is CheckMate 577 which demonstrated increased patient overall survival by 11 months when given a PD-1 inhibitor (nivolumab) as an adjuvant treatment for locally advanced OC following neoadjuvant chemotherapy and surgery [6], with a fraction of patients showing better response to the therapy than others [21–23]. This has been further improved by addition of anti-PD-L1 (durvalumab) in the MATTERHORN study where neoadjuvant chemoimmunotherapy demonstrates improved pathological complete response rates compared to chemotherapy alone [24]. Advancement has been more limited for patients with inoperable or metastatic disease potentially due to high rates of primary therapeutic resistance − trials of immunotherapy in combination with chemo/chemoradiotherapy are ongoing [25].

The last decade has seen exponential growth in our knowledge of OC genomics, especially its mutational landscape [26] but clinical success from consequent therapies has been modest [27]. Hence, there is greater appreciation that often-overlooked tumour cell-extrinsic factors such as the tumour microenvironment (TME), especially the stroma, may regulate tumour-permissive properties in OC that remain poorly characterised such as early lymph node involvement [28], high rates of systemic recurrence and poor therapeutic response. This includes a closer study of cancer-associated fibroblasts (CAFs), a dynamic and heterogenous [29] group of cells that form the largest cellular component of the stroma, known for modulating protumorigenic features through protein expression and secretion [30,31]. Notably, CAFs modify the tumour matrix microenvironment as master regulators of the extracellular matrix (ECM) [32].

**Fig. 1:**
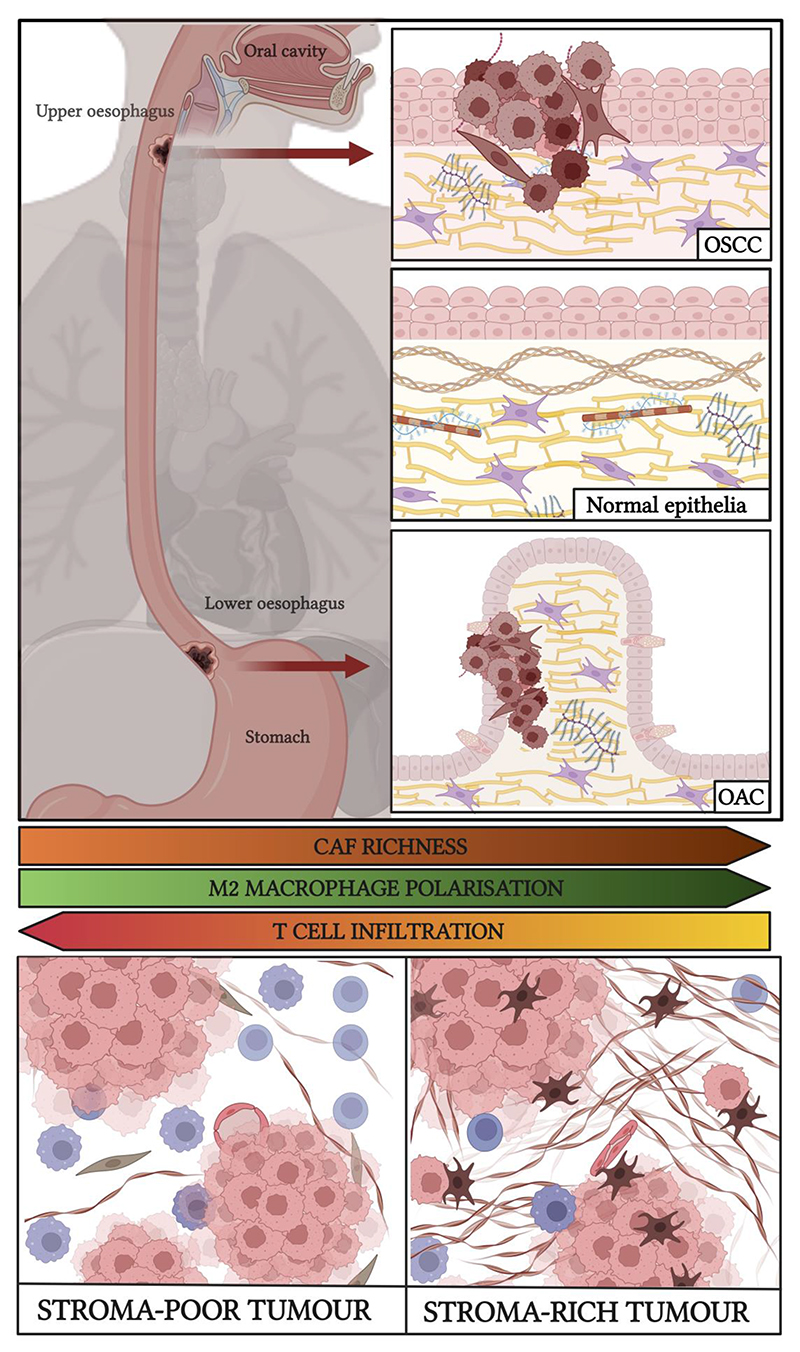
Stromal heterogeneity in oesophageal cancer. The normal oesophageal mucosa is made of stratified squamous cell epithelium. Oesophageal squamous cell carcinoma (OSCC) arises from the upper two-thirds of the oesophagus whereas oesophageal adenocarcinoma (OAC), associated with Barrett’s oesophagus, occurs in the lower third. Stroma-high tumours in OC, especially at the late stage of disease, are associated with poorer prognosis with reduced anti-tumour immune infiltration. OAC, oesophageal adenocarcinoma; OSCC, oesophageal squamous cell carcinoma. Created in BioRender.com.

Our developing understanding of CAFs and the ECM may address urgent unmet needs in OC [33–35] such as: (a) identifying biomarkers for screening and risk stratification, and (b) finding new therapeutic vulnerabilities to complement current treatment or uncover novel therapies. CAFs and the ECM share a bidirectional relationship during tumorigenesis that is tightly regulated by physical, mechanical and biochemical properties with clinicopathological consequences. This review will focus on their synergistic relationship with the goal of identifying molecular and pathologic clues that may help solve diagnostic and therapeutic challenges in OC by looking through a stromal lens (Fig. 1).

## Tumour stroma in oesophageal cancer

The TME is a heterogenous and complex ecosystem consisting of cellular and non-cellular compartments. Cellular components of the tumour stroma comprise of resident and invading cells such as fibroblasts, mesenchymal cells, immune cells, endothelial cells, pericytes and adipocytes [36], and the non-cellular compartment consists of the ECM, secreted and lymphatic molecules [37,38]. Although cancer was previously considered to be a cell-autonomous process, increasingly, evidence suggests that a favourable TME is crucial for tumour initiation and progression [39], in keeping with Paget’s “seed and soil” hypothesis [40]. Given the heterogeneity and plasticity of stromal components in the solid cancer TME, it is likely that different subpopulations of cellular and non-cellular components predominate in different stages of OC development and can exhibit both protumorigenic and antitumorigenic characteristics.

### Fibrosis and desmoplasia

Fibrosis describes the gradual build-up of intratumoural and peritumoural ECM components. This process exists within a spectrum whereby different degrees of stromal deposition have been identified not only between different patients but also within the same tumour sample, forming stromal rich and stromal poor areas [41]. Desmoplasia is a severe form of fibrosis secondary to an insult with the deposition of large amounts of dense stroma, which is well documented in solid tumours such as breast, colorectal and pancreatic cancers [42,43]. Desmoplastic stroma primarily consists of collagen and other connective tissue elements, forming a border between the tumour cells and the healthy tissue which result in a fibrotic niche. The tumorigenic factors that initiate and perpetuate the insults that lead to desmoplasia in solid cancers remain poorly characterised. It largely remains unclear how cancer cells interact with nearby fibroblasts to initiate fibrosis that results in scarring and subsequent fibrous ECM deposition, but chronic inflammation, hypoxia and nutrient deprivation likely play a key role to foster an environment that encourages ECM accumulation and subsequent pathogenic fibrosis.

Although studies have primarily investigated the detrimental role of fibrosis in OC [44–46], antitumorigenic properties of this process cannot be overlooked. It is likely that during early stages of tumorigenesis, stiffening and scarring of surrounding stromal tissue has important tumour-restraining properties by restricting the motility and invasion of cancer cells [47]. In later stages, ungoverned fibrosis, especially desmoplasia, may create a protective tumour niche which provides a physical barrier that prevents the access of tumour-restrictive components such as immune cells and anti-cancer therapies [48]. Hence, cancer cells that adapt to remain motile and retain their ability to invade, migrate and metastasise despite being encapsulated by a stiff stroma can hijack this initial homeostatic process to further proliferate [49]. There may even be a paradoxical stromal dynamic during tumorigenesis whereby stiffness may be desirable during tumour growth and progression, but tumour budding, and metastasis requires uncaging of tumour cells, loss of attachment and increased motility. Indeed, CAFs and associated fibrous ECM can initially encapsulate cancer cells to evade detection and elimination [50]. Later, CAFs can steer ECM fibers to re-orientate which encourages cancer cell movement [51], eventually leading to metastatic spread. It is therefore unsurprising that anti-fibrotic agents have been associated with better outcomes in patients with desmoplastic solid cancers [52].

### Stromal density in oesophageal cancer

Extensive histopathologic analysis of OC and gastric cancers has previously highlighted their densely fibrotic nature, with up to 90 % of the tumour mass consisting of stroma alone [53–57]. In the UK, fibrosis and desmoplasia are not features that are currently routinely reported in formal pathological analysis of OC tumour samples from biopsy or oesophagectomy specimen. Additionally, radiological estimation of tumour-stromal content and stiffness in gastrointestinal cancers is still in its infancy [58]. Nonetheless, tumour-stroma ratio (TSR) has been identified as a prognostic histological characteristic in OC, and both overall and disease-free survival rates are significantly improved in OC patients whose tumours were stroma-poor compared to those with stroma-rich disease [59–61]. However, despite robust clinical evidence, this prevailing consensus of fibrotic solid cancers has been challenged by newer bioinformatic and digital pathologic analyses, which have found mixed results for both tumour-stroma density and its prognostic significance in gastrointestinal cancers [62,63]. Micke et al. (2021) quantified the tumour stroma in 16 solid cancer types, including gastro-oesophageal junction adenocarcinoma, and found that the stromal fraction was highly variable, ranging from less than 25 % to over 70 % [62]. The clinical utility of the fibrous stroma was ambiguous as a higher stroma fraction was associated with shorter survival in some cancers, but not in others.

## The extracellular matrix in oesophageal cancer

The ECM consists of fibrillar and nonfibrillar collagens, glycoproteins, proteoglycan, basement membrane and other components that can be dysregulated during tumorigenesis. Pearce et al. (2018) found that solid tumours produce a common matrix response with 22 widely dysregulated ECM protein chains including collagen alpha-1(I) (*COL1A1*), collagen alpha-6(VI) (*COL6A6*), collagen alpha-1 (XI) (*COL11A1*), collagen alpha-1(XV) (*COL15A1*), fibronectin (*FN1*) and versican (*VCAN*), strongly associated with poorer overall survival, which also applies to OC [64]. This dysregulated matrix, which correlated with tumour stiffness, was found mainly in areas occupied by alpha-smooth muscle actin-positive (α-SMA +ve) and fibroblast activation protein-positive (FAP +ve) cells, alongside lymphocytes and macrophages. Additionally, cancer-specific ECM-signatures have been identified in breast [65], ovarian [66] and gastrointestinal cancers [67], associated with chemotherapy resistance and poorer survival, which is likely also the case with OC.

The key players in dysregulated ECM remodeling of solid cancers include cancer cells, CAFs and tumour-associated macrophages (TAMs), with minor contributions from adipocytes [68] and other immune cells. Cancer cells can directly [37] or indirectly [69,70] produce and degrade ECM, and cancer cell-derived ECM correlates with poor patient outcomes. Although OC-specific cancer secretome is not well characterised, Robinson et al. (2019) reported that most secreted proteins in solid cancers, including OC, perform functions involved in ECM turnover [71]. TAMs also significantly contribute to ECM production [72] and degradation [73], whereby TAM depletion leads to reduced collagen deposition [74]. ECM degradation by TAMs is thought to promote angiogenesis, which may further permit tumour spread.

Most importantly, CAFs are major regulators of the ECM, with crucial roles in ECM deposition, remodeling and breakdown, through direct [75] and exosomal secretion [69]. CAF-mediated production of ECM is associated with unique, cancer-specific phenotypes. In the absence of cancer cells or TAMs, models of ECM recapitulate stromal biomechanical properties of cancer, as ECM shapes and structures the TME [76,77]. Dysregulated ECM proteins secreted by CAFs in OC have been listed in Table 1. Accordingly, *in vivo* CAF depletion completely abrogates stromal dysregulation necessary for tumour persistence [78]. It is therefore imperative that CAF and ECM components are analysed together as distinct CAF subpopulations likely secrete and modulate specific ECM signatures in the OC TME.

## Identifying cancer-associated fibroblasts in oesophageal cancer

In keeping with poor responses identified in stromal rich tumours, OCs with high infiltration of CAFs have poorer chemotherapy [110] and immunotherapy response with overall worse prognosis [111]. CAFs, which encapsulate, marginate and infiltrate tumour cells, can express dysregulated proteins including growth hormones and cytokines through direct or exosomal secretion [112], which promote protumorigenic properties such as immune exclusion [113]. Although transcriptomic and spatial profiling techniques predominate the current CAF landscape due to lineage and functional complexity, earlier studies looking into distinct, disease-specific CAF markers in OC have highlighted CAF biology, canonical signaling pathways and stromal targets, often at the cost of overlooking heterogeneity. These markers which are often characterised at the protein-level, as highlighted in Table 2, are still used for sorting and classifying CAFs in the TME for more advanced analyses.

A pan-cancer CAF marker remains elusive and podoplanin (*PDPN*) in pancreatic ductal adenocarcinoma (PDAC) may be the only consistent, disease-specific CAF marker identified in cancer to-date [114,115]. Similar success has not been recapitulated in OC as podoplanin expression is variable with significant overlap between the tumour cell, normal cell and stromal expression. Although Tanaka et al. (2015) and Schoppmann et al. (2013) reported that podoplanin+ CAFs were associated with poorer prognosis in OC, these were inconsistent with findings from Chuang et al. (2014), pending further validation and mechanistic studies [116–118]. Evidence in OC is similarly low for other “pan-CAF markers” such as fibroblast-specific protein 1 (*FSP1*), thy-1/CD90 (*THY1*) and basigin/ CD147 (*BSG*).

αSMA and FAP are reliable CAF markers in both OSCC and OAC, closely associated with the myofibroblastic CAF (myCAF) phenotype. αSMA +ve myCAFs are abundantly present at the tumour invasive front in OSCC, resulting in chemoresistance with worse prognosis [119]. Similarly, αSMA +ve myCAFs also control invasiveness in OAC through modulation of the matricellular protein, periostin (*POSTN*) [98]. In comparison, FAP +ve CAFs in OC have mainly been implicated in lymph node metastasis. FAP +ve CAFs in OSCC can promote tumour cell invasion into the surrounding lymph nodes through CCL2, IL6 and CXCL8 secretion inducing M2-macrophage polarisation [120]. Selective FAP +ve CAF ablation in OAC in mouse models improved tumour cell response to 5-fluorouracil [121], suggesting a role of these stromal cells in therapy resistance.

Other CAF markers such as platelet-derived growth factor receptors-α/β (*PDGFRα/β*), Twist-related protein 1 (*TWIST1*) and vimentin (*VIM*) have shown promise in OSCC, but not OAC. Of note, although PDGFRβ is increasingly being used as a pericyte marker, it is also expressed in subsets of CAFs. Expression of both PDGFRα and PDGFRβ is higher in OSCC compared to OAC [122] and PDGFRα +ve CAFs are often associated with an inflammatory CAF (iCAF) phenotype [123]. TWIST1 is an embryogenic transcription factor that is involved in epithelial-to-mesenchymal transition (EMT) in OSCC, which is associated with higher invasiveness and tumorigenicity [124,125]. VIM is also another EMT marker on CAFs that has recently been implicated more widely in CD8+ *T* cell exclusion in OSCC [126]. Given the plasticity of CAFs and abundance of potential markers, further studies characterising CAFs in OC are required, particularly in OAC where there remains significant potential to improve our current knowledge. As CAFs have a critical role in mediating chemo- and immuno-resistance, understanding and targeting these populations could result in significant therapeutic gains.

## Cancer-associated fibroblast-mediated extracellular matrix remodelling in oesophageal cancer

### Oesophageal squamous cell carcinoma

#### Myofibroblastic cancer-associated fibroblasts

CAF subpopulations have been more extensively profiled in OSCC compared to OAC, with myCAFs and iCAFs consistently identified. Jia et al. (2023) reported that myCAFs were the most abundant type of fibroblasts in OSCC, highly expressing dysregulated ECM proteins such as collagen chain alpha-1(I) (*COL1A1*), collagen triple helix repeat-containing protein 1 (*CTHRC1*), interstitial collagenase (*MMP1*), stromelysin-3 (*MMP11*) and periostin (*POSTN*) [142]. Similarly, Zhang et al. (2021) found that myCAFs in OSCC participate in EMT pathways and express ECM proteins like MMP1, MMP11 and transgelin (*TAGLN*) [143]. Interestingly, both studies also investigated the origin of myCAFs in OSCC and hypothesised that pericytes could be a potential precursor cell type. Transgelin expression was also found to be upregulated in OSCC myCAFs in another transcriptomic study, the presence of which is associated with poorer patient survival [144]. Transgelin is a TGF-β-inducible protein normally involved in calcium interactions and contractile properties of the cell. It has been shown to promote lung cancer progression via CAF activation but its role in OSCC is unknown [145].

Besides transgelin, myCAFs also upregulated other proteins such as collagen chain alpha-1(I), MMP11 and periostin. Collagen alpha-1(I) chain has been reported to be highly expressed in OC stroma, and its upregulation has been associated with cancer cell proliferation and invasion [146]. Although MMP11 is thought to play a dual role in cancers [147], its expression in OSCC is typically associated with increased invasiveness and poorer prognosis. In a multicohort gene-based analysis, Jiali et al. (2020) reported that matrix metalloproteases were implicated in stromal activation with upregulation in OSCC correlating with poorer prognosis [89]. myCAFs have additionally been reported to upregulate expression of periostin. Periostin plays a central role in extracellular matrix organisation, as it acts as a scaffold for extracellular matrix proteins, including fibronectin and tenascin-C, as well as interacting with collagens to enable collagen crosslinking [148]. In keeping with this central role, periostin has been implicated in OSCC pathology. For example, Miyako et al. (2024) reported that periostin secreted by CAFs in OSCC increased tumour survival, growth, and migration through the AKT-ERK pathway via ADAM17 activation [149] with resulting poor postoperative outcomes [97].

#### Inflammatory cancer-associated fibroblasts

iCAFs in OSCC which show increased expression of PDGFRα actively secrete inflammatory cytokines such as IL6 [142], CXCL8 and CXCL12 [144,150]. They have been predicted to participate in immune cell recruitment and may potentially orchestrate antitumorigenic inflammatory response. Guo et al. (2022) reported that the iCAF population is restricted to the stromal regions, while myCAFs were significantly enriched in both tumour and stromal regions [151]. This finding is in-keeping with spatial profiling studies in PDAC [152] and colorectal cancer (CRC) which have shown the antagonistic relationship between tumour-permissive myCAFs and tumour-suppressive iCAFs, separated by proximity to tumour cells. In CRC, the tumour-proximal TGF-β-responsive myCAF and tumour-distal BMP signaling-driven iCAF niches are maintained by a gradient of growth factors in the TME [153]. However, this is closely related to the normal embryogenic development of the colonic mucosa and similar growth factor or cytokine gradient-driven formation of CAF subpopulations and subsequent spatial niches have not been demonstrated in OC. Additionally, our understanding in both OAC and OSCC lacks how the spatial configuration of distinct CAF subpopulations shape the landscape of ECM, which may have implications on immune cell infiltration, angiogenesis, tumour cell invasion and metastasis. This could partly be accounted for by the practice of neoadjuvant therapy in OC, which is not commonly offered for CRC. Therefore, whole resection specimens are less abundantly available for pathological interrogation in OC.

#### Other cancer-associated fibroblast subpopulations

Other CAF subpopulations that have been identified in OSCC include antigen-presenting CAFs [82,142,144], which were first characterised in PDAC in 2019 [114]. These apCAFs have a high expression of genes related to MHC class II presentation such as HLA-DRA, HLA-DRB1 and CD74, with negligible levels of the costimulatory genes (CD80 and CD86) which paradoxically dampens anti-tumour immune response compared to professional antigen-presenting cells [81]. These cells have been hypothesised to act as a competitive decoy for the tumour specific CD4+ *T* cells in an environment where tumour neoantigens are limited. Similarly, complement-expressing CAFs present in OSCC and OAC upregulate broadly specific proteases in the complement system, such as C3 and C7, which may be used for protumorigenic functions as opposed to complement activation and complement-dependent cytotoxicity. Other studies have also reported more sporadic CAF subpopulations in OSCC such as cystatin-SN-positive (CST1 +ve) CAFs, which have a strong ECM modulatory activity [82]. CD73+ CAFs are another rarely reported CAF subpopulation. CD73 plays a major role in the adenosine pathway by catalysing the conversion of AMP to adenosine. Extracellular adenosine in the TME has been shown to support immunosup-pression and promote cancer cell metastasis [154]. Interestingly, we and others reported that ectonucleotide pyrophosphatase/phosphodiesterase family member 1 (ENPP1), an ectonucleotidase in the TME which hydrolyses cGAMP to AMP in a parallel pathway, is upregulated in chromosomally unstable cancers [154]. However, the role of ENPP1 on fibroblasts and how this may contribute to intracellular adenosine and immunosuppression is currently unknown.

#### Oesophageal adenocarcinoma

In OAC, the heterogeneity of CAFs has been underreported with limited transcriptomic studies identifying distinct CAF subpopulations in this disease. Croft et al. (2022) identified seven CAF subpopulations, including myCAFs, adipogenic CAFs (aCAFs) and complement-expressing CAFs, two of which were distinct to OAC compared to normal adjacent tissue [84]. Multiple key points stand out from this study which are: (a) several defining markers of CAF subpopulations were dysregulated ECM proteins including collagen alpha-2(I) chain (*COL1A2*) and microfibrillar-associated protein 5 (*MFAP5*) [155], (b) two subpopulations of myCAFs were identified with defining markers of myosin-11 (*MYH11*) and metalloreductase STEAP4 (*STEAP4*) and (c) all fibroblast subpopulations had an expression profile enriched in matrix proteins such as collagen chains alpha-2(I) (*COL1A2*) and alpha-1(III) (*COL3A1*), lumican (*LUM*) and SPARC (*SPARC*).

**Fig. 2:**
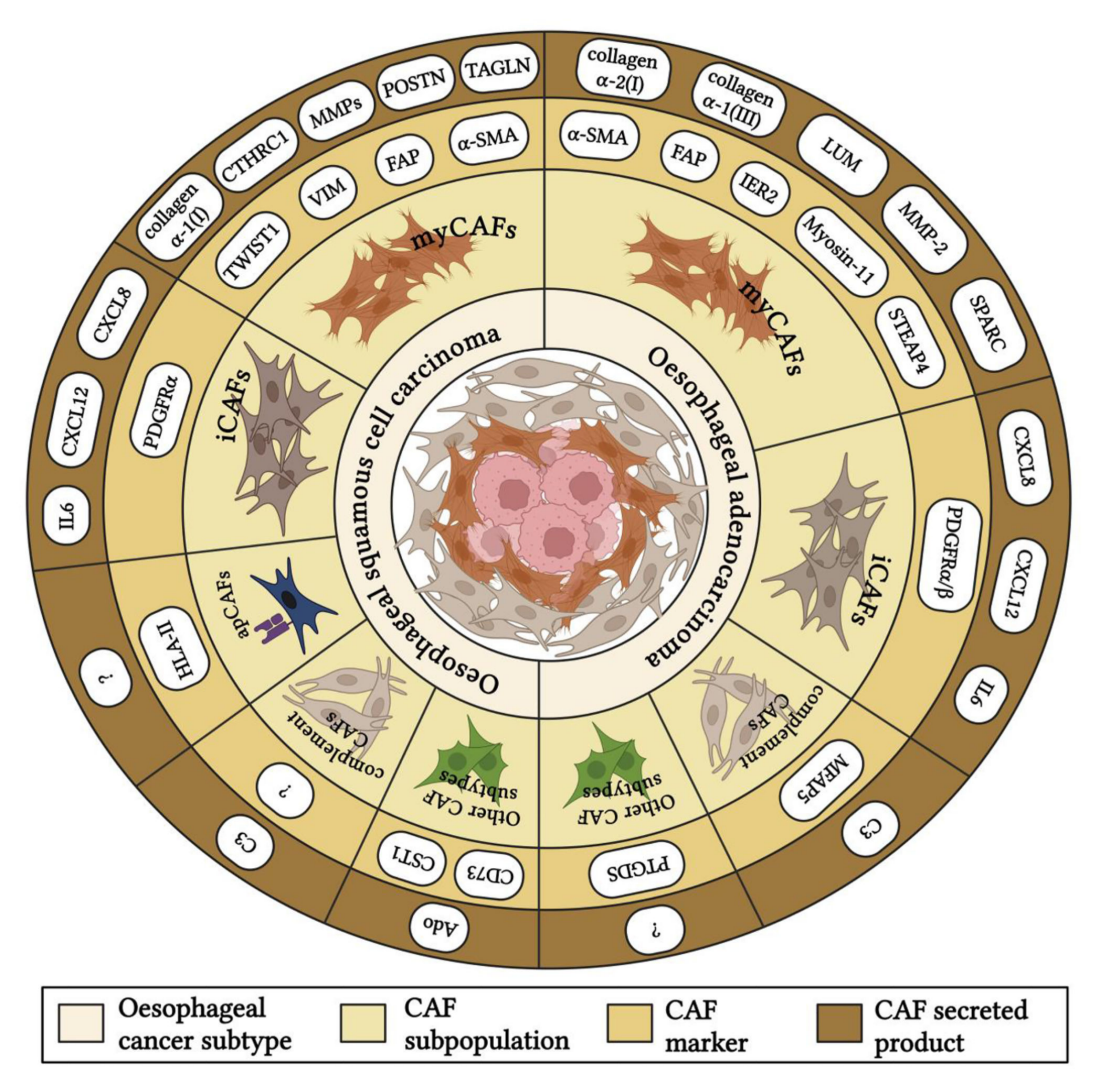
Cancer-associated fibroblast (CAF) subpopulation in oesophageal cancer (OC). Schematic representation of CAF subpopulations identified for each OC subtype alongside the expression marker and secreted proteins, which are mainly extracellular matrix (ECM) components. Ado, adenosine; α-SMA, alpha smooth muscle actin; C3, complement component 3; CST1, cystatin 1; CTHRC1, collagen triple helix repeat containing 1; FAP, fibroblast activation protein; HLA, human leukocyte antigen; IER2, immediate early response protein 2; LUM, lumican; MFAP5, microfibril associated protein 5; MMP, matrix metalloprotease; PDGFR, platelet-derived growth factor receptor; POSTN, periostin; PTGDS, prostaglandin D2 synthase; SPARC, secreted protein acidic and rich in cysteine; STEAP4, six trans-membrane epithelial antigen of prostate protein 4; TAGLN, tangelin. Created in BioRender.com.

Although these observations support the claim that CAFs modulate the ECM in OAC, further studies are required to confirm and identify more specific and targetable CAF subpopulations with distinct matrix expression profiles. We know that there is a cluster of distinct matrix markers in OAC that was elucidated by Naeini et al. (2023), which are associated with poor immune cell infiltration, higher nodal involvement after surgery, poorer response to neoadjuvant chemotherapy and worse overall survival [88]. They found that these immune-suppressed tumour samples had upregulated A disintegrin and metalloproteinase with thrombospondin motifs 4 (*ADAMTS4*), collagen chains alpha-1(IV) (*COL4A1*) and alpha-2 (IV) (*COL4A2*), stromelysin-1 (*MMP3*) and osteopontin (*SPP1*), but the source of these dysregulated proteins, which are likely a CAF subpopulation, remains uncharacterised (Fig. 2).

## Therapy resistance mediated by oesophageal cancer stroma

### Chemoresistance

Conventional treatment for OC typically includes radiotherapy, chemotherapy, and surgery. Therapeutic resistance is often attributed to mechanisms that include changes in drug targets, drug efflux, circumvention of apoptosis, DNA damage repair, and stemness [reviewed in [156,157]]. Activated fibroblasts are also likely accomplices. *In vitro*, OSCC cell lines grown in conditioned media from CAFs treated with cisplatin had enhanced viability and reduced sensitivity to chemother-apeutics via PAI-1-mitigation of reactive oxygen species accumulation, thereby reducing oxidative stress [158]. This was also demonstrated in OSCC PDX models where crosstalk between OSCC cells and CD147+ CAFs via cancer-secreted S100A8 induced transition of these fibroblasts to a myCAF phenotype, via the CD147-RhoA-ROCK-MLC2-MRTF-A pathway, indicated by upregulation of αSMA coupled with type I collagen. These activated myCAFs reciprocated by conferring chemoresistance to OSCC when co-cultured *in vitro* upon treatment with chemotherapeutics via activation of integrin-mediated anti-apoptotic pathways [70].

Additionally, chemotherapy may also contribute to increased myCAF populations in OC. Normal fibroblasts exposed to cisplatin and fluorouracil, a common chemotherapeutic combination for OC treatment, significantly upregulated FAP and αSMA expression, markers of a transition to a myCAF-like state, by a 2−4-fold factor [159]. Tissue from OSCC patients with a higher proportion of fibroblasts with an activated morphology *i.e*., enlarged with a branching cytoplasm as well as being multi-nucleated, showed greater tumour-associated macrophage infiltration as well as microvascular density [136], both linked to poor prognosis in OSCC [160,161]. Taken together, these findings suggest that chemotherapy may cause fibroblasts to transition to a more pro-tumour, myCAF-like phenotype and promote fibroblast-mediated chemoresistance, adversely impacting overall survival.

### Radiotherapy resistance

Similar reports point to CAFs as a source of radiotherapy resistance in OC. CAFs with upregulation of CXCL1, a chemokine implicated in cancer progression can induce radiation resistance [162]. *In vitro* investigations reported that CAF-secreted CXCL1 could augment DNA damage repair in OC cells exposed to radiation via the MEK/ERK pathway, the effects of which were nullified *in vivo* using CXCL1 blocking antibodies [163]. Type I collagen is also implicated as an accessory to CXCL1 in promoting radiation resistance. Similarly, the resistance mechanism involves DNA damage repair in response to radiation reportedly via the route of FAK/c-MYC/CHK1 signalling, triggered by integrin interactions with type I collagen [164].

In addition, conditioned media from OSCC cells grown on type I collagen promoted fibroblast migration and upregulation of myCAF markers. Radiation exposure also appears to be a stronger trigger of myCAF induction than cisplatin and fluorouracil as indicated by a 4−10-fold change in αSMA and FAP upregulation. Consequently, OC cells cultured in conditioned media from radiation-activated fibroblasts had reduced sensitivity to X-ray irradiation [159]. Since myCAFs are the primary type I collagen producers in the TME, this can create a positive feedback loop of perpetual myCAF recruitment and reduced radiation sensitivity, which could be detrimental to patients whose tumours have a high stromal content.

## Stromal-targeting strategies in oesophageal cancer

CAFs and the ECM can augment cancer progression, showing that not only does the nature of the cancer cells have to be considered, but the tumour-permissive stromal compartment should also be considered when determining the course of treatment. Thus, the roles of CAFs and ECM in tumour progression and therapeutic resistance make them attractive targets to supplement therapies that directly target cancer cells. Strategies to modulate CAF activity and ECM architecture in favour of tumour suppression typically attempt to block crosstalk between cancer cells, deplete CAFs, or ECM degradation.

Phototherapy, which uses light-activated drug conjugates, has recently emerged as a promising avenue for selectively targeting FAP+ve CAFs in OC while minimizing off-target effects such as bone marrow toxicity, osteotoxicity, muscle damage and cachexia [165]. Tumour stroma with high FAP expression has been linked to poorer outcomes [166], making it a suitable candidate for targeted therapy. Anti-FAP recombinant antibody conjugated to a photoreactive dye, IR700, has been shown to discriminately eliminate FAP +ve CAFs *in vitro* in a dose-dependent manner − normal fibroblasts and OC cancer cells were unaffected with *in vivo* models having no observable side effects [141]. Targeting FAP in this manner has been coupled with IR700-conjugated panitumumab and trastuzumab targeting OC cells, with greater efficacy compared to single-target treatment and no observable off-target effects. In immune-competent models, the elimination of FAP +ve CAFs aided in amplifying CD8+ lymphocyte infiltrate, resulting in enhanced tumour suppression. Given the extent to which the TME hinders the efficacy of immunotherapies, targeting FAP +ve CAFs in this manner makes this a viable combination candidate for immunotherapies against particularly immune-cold OC cancers with high stromal content. An interesting effect of FAP+ve CAF depletion was a reduction in TGF-β secretion, which could mitigate paracrine activation of neighbouring fibroblasts into myCAFs. Although older studies have suggested off-tumour/on-target complications associated with FAP ablation due to muscle and bone marrow expression, recent selective targeting in animal models have been more successful [141].

**Fig. 3:**
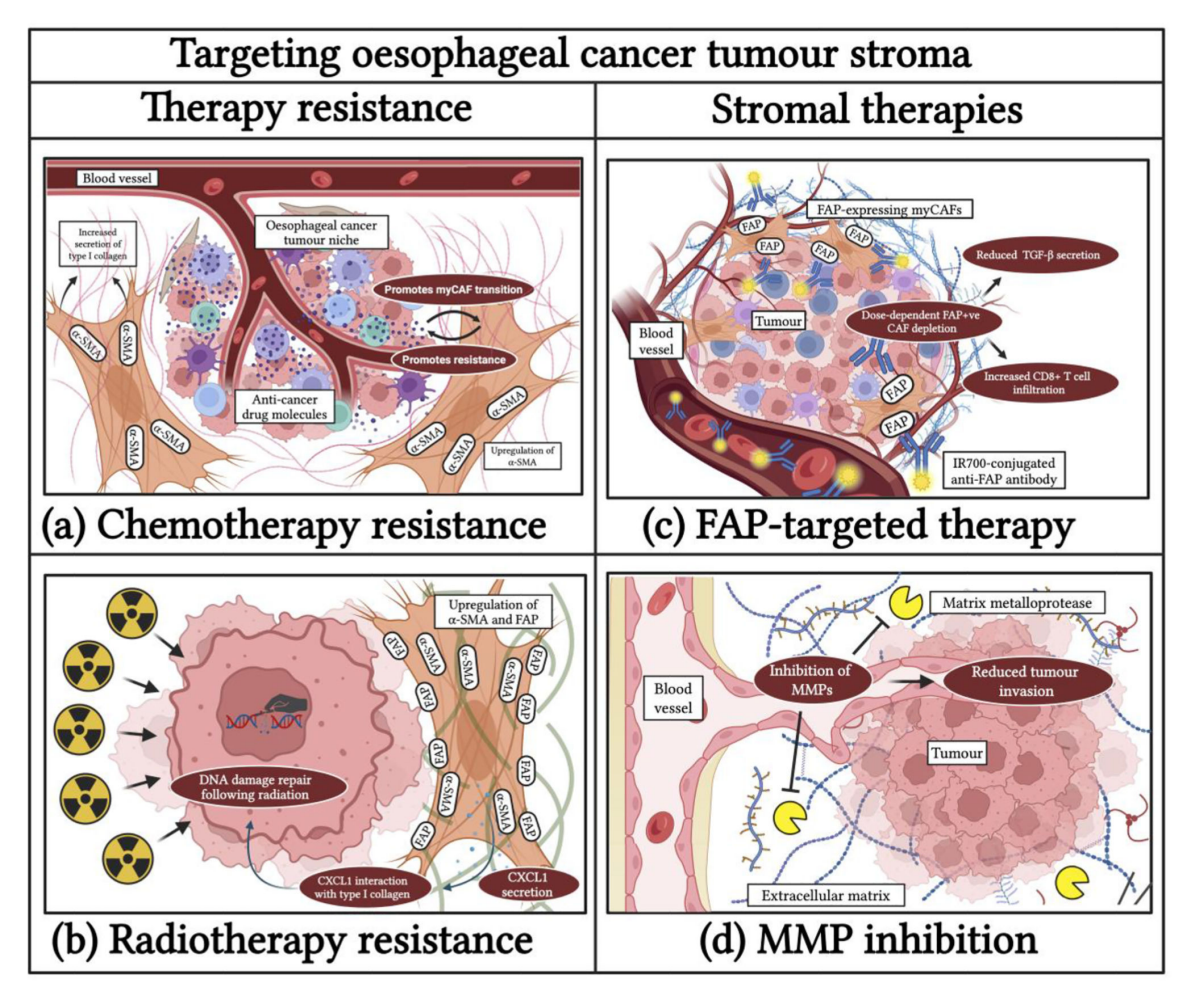
Targeting oesophageal cancer tumour stroma. Cancer-associated fibroblasts (CAFs) and the extracellular matrix (ECM) play a role in oesophageal cancer therapy resistance, and targeting their components form part of novel stromal therapies. (a) Chemotherapy resistance. (b) Radiotherapy resistance. (c) FAP-targeted therapy. (d) MMP inhibition. α-SMA, alpha smooth muscle actin; CXCL1, chemokine (C-X-C motif) ligand 1; FAP, fibroblast activation protein; IR700, near-infrared (NIR) fluorescent dye with a wavelength of around 700 nm; MMP, matrix metalloprotease. Created in BioRender.com.

Other strategies involve targeting acellular stromal components that, if dysregulated, can support tumour progression. Matrix metalloproteinases (MMPs), a group of enzymes responsible for ECM remodelling by degrading matrix proteins, can contribute to cancer invasion into surrounding tissue and metastasis. *In vitro*, siRNA knockdown of MMP14 limited the invasive potential of OSCC cells through a Matrigel matrix [167]. In the clinical setting, a phase III, randomized, double-blind clinical trial sought to assess the efficacy of andecaliximab, an MMP9 inhibitor, in combination with fluorouracil. Results showed that although the combination did not induce significant adverse effects, overall survival was not improved compared to fluorouracil monotherapy. Even though patients treated with andecaliximab alone had a higher objective response rate over the placebo group, this did not lead to an improvement in progression-free or overall survival [168] (Fig. 3).

## Future directions

The complex interplay between CAFs and the ECM in OC impacts tumour progression, invasion of surrounding tissue, angiogenesis and metastasis. With greater appreciation of stromal heterogeneity in OC, we are working towards identifying distinct, targetable CAF subpopulations in OSCC and OAC that are responsible for modulating a dysregulated ECM profile in the tumour stroma. Stromal populations are complex in that they play both anti- and pro-tumourigenic roles, and broad targeting of this population carries risks, potentially greater in early stages of tumourigenesis. Therefore, it is important to consider all stages of disease when studying OC, including premalignant lesions such as Barrett’s oesophagus in OAC. Given the plasticity of CAFs and the ECM, only by better characterising both protumorigenic and anti-tumorigenic functions of distinct stromal subpopulations would it be possible to design therapies that specifically target components that support tumour development.

Additionally, besides cell-intrinsic differences between OSCC and OAC such as mutational signatures and genomic abnormalities that leads to disparities in lymphovascular invasion, metastasis, recurrence after surgery and therapy response, disease-specific stromal remodeling likely also plays a role. It is important to highlight that although CAF-ECM interactions in OC are generally understudied, there is fewer studies investigating CAF-ECM heterogeneity and signatures in OAC compared to OSCC. More disease-specific inquiry into the tumour stroma is required for OAC, using methods that take into consideration the heterogeneity and functional complexity of CAFs and the ECM. This can be complemented by studies that investigate origins, intracellular molecular pathways and plasticity of CAFs and the ECM in OC. These efforts may help us overcome barriers in translating stromal-targeting agents into the clinics for OC which are the lack of specificity, unexpectedly low efficacy and the presence of off-target side effects. Understanding tumour-stromal interactions and crosstalk throughout cancer development is essential to develop and improve future treatment strategies for OC.

## Figures and Tables

**Table 1:** List of selected secreted proteins overexpressed in OSCC and OAC CAFs.

*Secreted protein name*	*Encoding gene*	*Function in normal physiology*	*Possible pathological role*	*Histologic subtype*	*Study technique*	*Source*
**Collagens** [79]						
Collagen chain alpha-1(I)	*COL1A1*	Forms type I collagen	TME scaffolding and immune exclusion	OSCC	scRNA seq	[80−82]
Collagen chain alpha-2(I)	*COL1A2*	Forms type I collagen	Tumour growth and progression	OAC OSCC	Bulk RNA seq, sc RNA seq IHC	[83,84] [85]
Collagen chain alpha-1(III)	*COL3A1*	Forms type III collagen	Tumour aggressiveness	OAC OSCC	scRNA seq, IHC scRNA seq, bulk	[84,85] [86,87]
Collagen chains alpha-1 and 2(IV)	*COL4A1* and *COL4A2*	Forms type IV collagen	Tumour invasion and metastasis	OAC	RNA seq, IHC MS	[88]
Collagen chain alpha-1(V)	*COL5A1*	Forms type V collagen	Proliferation and migration	OSCC	scRNA seq	[81]
Collagen chain alpha-2(VI)	*COL6A2*	Forms type VI collagen	Tumour growth and progression	OSCC	scRNA seq	[89]
Collagen chain alpha-1(XI)	*COL11A1*	Forms minor fibrillar collagen	Tumour proliferation, migration and invasion	OAC	IHC	[90]
**Proteoglycans**						
Lumican	*LUM*	Collagen fibrillogenesis	Cancer growth and invasion	OSCC	IHC	[91,92]
Versican	*VCAN*	Mitotic spindle fibre organisation	Proliferation and angiogenesis	OSCC	IHC	[92]
**ECM glycoproteins**						
Fibronectin	*FN1*	Cell adhesion and matrix assembly	Invasion and metastasis	OSCC	IHC, WB	[93,94]
Laminin subunit alpha-1	*LAMA1*	Cell attachment, migration and organisation	Remains poorly characterised	OSCC	RT-qPCR, IHC	[95]
Microfibrillar-associated protein 5	*MFAP5*	Fibrosis	Matrix remodeling	OAC	scRNA seq	[84]
Lactadherin	*MFGE8*	Mucosal healing and phagocytosis	Angiogenesis and tumour proliferation	OSCC	scRNA seq, IF, IHC, WB	[96]
Periostin	*POSTN*	Wound healing and ECM deposition	Invasion and metastasis	OSCC OAC	WB IHC	[91,97] [98]
SPARC	*SPARC*	Cell adhesion and interaction	Tumour growth and metastasis	OSCC	TMA, IHC	[90]
Osteopontin	*SPP1*	CNS homeostatic sensor	Tumour growth and migration	OSCC	WB	[99, 100]
Tenascin	*TNC*	Induced during embryogenesis and wound healing for cell migration	Cancer progression, angiogenesis and metastasis	OSCC	IHC	[101]
Thrombospondin-2	*THBS2*	Cell proliferation and adhesion	Cell migration and invasion	OSCC	scRNA seq	[102]
**ECM regulators** A disintegrin and metalloproteinase with thrombospondin motifs 4	*ADAMTS4*	Extracellular protease	Tumour growth and angiogenesis	OAC	scRNA seq	[88]
Interstitial collagenase	*MMP1*	ECM remodeling	Lymph node invasion	OSCC	IHC, WB	[103]
Matrix metalloproteinase-9	*MMP9*	ECM remodeling	Tumour progression and invasion	OSCC	IHC	[104, 105]
Procollagen-lysine,2-oxoglutarate 5-dioxygenase 2	*PLOD2*	Collagen cross-linking	Cancer stemness	OSCC	scRNA seq	[106]
**ECM-affiliated/associated proteins**						
Collagen triple helix repeat-containing protein 1	*CTHRC1*	Vascular remodeling and tissue repair	Cancer progression through angiogenesis and metastasis	OSCC	IHC, WB	[107]
Gremlin-1	*GREM1*	Embryological limb and kidney development	Fibrosis and angiogenesis	OSCC	IHC, WB	[108]
Mucin-1	*MUC1*	Lubricant and barrier	Immunosuppression	OSCC	RT-PCR, IHC	[109]

IHC, immunohistochemistry; MS, mass spectometry; RT-PCR, reverse transcriptase polymerase chain reaction; scRNA seq, single cell RNA sequencing; TMA, tissue microarray; WB, western blotting.

**Table 2:** List of important CAF markers identified in OSCC and OAC.

CAF marker	Expression			Localisation	Pathological role	Histologic subtype	Protein-level validation technique	Source
	Fibroblasts	Tumour cells	Normal cells					
αSMA	High	Low	Low	Cytosolic filament	Associated with invasiveness	OSCC OAC	IF, IHC, WB IF, IHC, WB	[119,127] [98,128]
FAP	High	Low	Variable	Surface glycoprotein	Lymph node metastasis	OSCC OAC	ELISA, IHC, IP, WB WB	[28,120, 129−132] [121]
FGFR2	High	Mixed	Mixed	Surface receptor	EMT	OSCC	IF, IHC, WB	[133–135]
FSP1	High	Low	Low	Cytosolic filament-associated	Unknown	OSCC	IHC	[136]
PDE5	Highest	Variable	Variable	Cytosolic hydrolase	Stromal contractility, invasion and chemoresistance	OAC	IHC, WB	[137]
PDGFRα/β	High	Low	Low	Surface receptor tyrosine kinase	Implicated in therapy resistance	OSCC	IF, IHC WB	[122,123,138,139]
Podoplanin	High	Variable	Low	Surface sialoglycoprotein	Tumour progression and metastasis	OSCC OAC	IHC IHC	[116,118] [117]
TWIST1	High	High	Low	Nuclear transcription factor	Increased tumorigenicity through migration and invasion, EMT	OSCC	IF, IHC, WB	[124,125]
VIM	High	High*	Low	Cytosolic filament	EMT	OSCC	IHC	[116,126,140]

αSMA, alpha smooth muscle actin; CAF, cancer-associated fibroblast; EMT, epithelial-to-mesenchymal transition; FAP, fibroblast activation protein; FGFR2, fibroblast growth factor receptor 2; FSP1, fibroblast specific protein 1; IF, immunofluorescence; IHC, immunohistochemistry; IP, immunoprecipitation; OAC, oesophageal adenocarcinoma; OSCC, oesophageal squamous cell carcinoma; PDE5, phosphodiesterase-5; PDGFRα/β, platelet-derived growth factor receptors-α/β; qRT-PCR, quantitative reverse transcription polymerase chain reaction; WB, western blotting.

* VIM becomes expressed in de-differentiating tumour cells as they assume a mesenchymal phenotype through EMT. However, VIM expression in mesenchymal cells and CAFs in the tumour microenvironment are the major sources of expression [141].

## Data Availability

No data was used for the research described in the article.
